# Phytoplankton Sources and Sinks of Dimethylsulphoniopropionate (DMSP) in Temperate Coastal Waters of Australia

**DOI:** 10.3390/microorganisms10081539

**Published:** 2022-07-29

**Authors:** Eva Fernandez, Justin R. Seymour, Katherina Petrou

**Affiliations:** 1 School of Life Sciences, University of Technology Sydney, Sydney, NSW 2007, Australia; eva.fernandez@alumni.uts.edu.au; 2 Climate Change Cluster, University of Technology Sydney, Sydney, NSW 2007, Australia; justin.seymour@uts.edu.au

**Keywords:** sulfur cycling, DMSP uptake, *Trichodesmium*, diatoms, size fractions, phytoplankton, east Australia

## Abstract

The ecologically important organic sulfur compound, dimethylsulfoniopropionate (DMSP), is ubiquitous in marine environments. Produced by some species of phytoplankton and bacteria, it plays a key role in cellular responses to environmental change. Recently, uptake of DMSP by non-DMSP-producing phytoplankton species has been demonstrated, highlighting knowledge gaps concerning DMSP distribution through the marine microbial food web. In this study, we traced the uptake and distribution of DMSP through a natural marine microbial community collected from off the eastern coastline Australia. We found a diverse phytoplankton community representing six major taxonomic groups and conducted DMSP-enrichment experiments both on the whole community, and the community separated into large (≥8.0 µm), medium (3.0–8.0 µm), and small (0.2–3.0 µm) size fractions. Our results revealed active uptake of DMSP in all three size fractions of the community, with the largest fraction (>8 µm) forming the major DMSP sink, where enrichment resulted in an increase of DMSPp by 144%. We observed evidence for DMSP catabolism in all size fractions with DMSP enrichment, highlighting loss from the system via MeSH or DMS production. Based on taxonomic diversity, we postulate the sources of DMSP were the dinoflagellates, *Phaeocystis* sp., and *Trichodesmium* sp., which were present in a relatively high abundance, and the sinks for DMSP were the diatoms and picoeucaryotes in this temperate community. These findings corroborate the role of hitherto disregarded phytoplankton taxa as potentially important players in the cycling of DMSP in coastal waters of Australia and emphasize the need to better understand the fate of accumulated DMSP and its significance in cellular metabolism of non-DMSP producers.

## 1. Introduction

Phytoplankton communities are shifting in response to climate change [1], with range shifts reported for many taxa [2,3,4,5,6]. Despite potential changes in species distributions, in-built phenotypic plasticity means that many phytoplankton have evolved physiological strategies to deal with the changing environmental conditions of the ocean [7,8]. One of these strategies is the ability to synthesize large amounts of dimethylsulfoniopropionate (DMSP), a strategy shown to protect cells against physiological stress, including changes in salinity, light, nutrient regime, or reactive oxygen production [9,10,11,12,13,14]. Among phytoplankton, the most prolific producers of DMSP include the haptophytes and dinoflagellates [15], whereas diatoms, chlorophytes, and cyanobacteria are generally considered low or non-producers [16,17]. Very recent work has also shown that some species of *Trichodesmium*, a diazotrophic cyanobacterium common to tropical waters, have the capacity to produce DMSP [17,18], suggesting a previously overlooked contribution to the marine sulfur cycle. Assuming DMSP synthesis provides protection and thereby offers some level of niche plasticity, and given that not all phytoplankton species produce DMSP, determining whether non-DMSP-producing phytoplankton can utilize DMSP from the dissolved pool in the marine environment could provide insight into the strategies these taxa employ to increase their resilience to changes in environmental conditions.

Once produced by the phytoplankton or bacterial cell, the un-utilized DMSP is released into the surrounding seawater, becoming biologically available to the marine community. This dissolved fraction (DMSPd) plays an important role in marine microbial ecology [19], influencing bacterial growth, competition, and sulfur cycling [20]. Generally, catabolism of DMSP is considered the domain of key marine bacterioplankton, occurring via two means: demethylation, through which DMSP is transformed to methanethiol (MeSH) to be utilized for energy and protein production [21,22], and DMSP cleavage, where DMSP is cleaved to the volatile gas dimethylsulfide (DMS) by lyases [19,23]. Identified Michaelis–Menten constants (Kms) for DMSP lyase range from 0.4 to 82 mM [24], and cleavage rates depend on the taxa, their sulfur demand, and their overall abundance [25,26,27]. Because these pathways are well studied, the role of DMSP metabolism and DMS production is largely attributed to heterotrophic bacterioplankton. However, lyase activity has been found in phytoplankton, including the haptophyte *Emiliania huxleyi* [27,28], dinoflagellates from the family of Symbiodiniaceae [29], and the prymnesiophyte *Phaeocystis* spp. [30], demonstrating that some phytoplankton also have the capacity to cleave DMSP to DMS. Moreover, it has been demonstrated that other non-DMSP-producing phytoplankton species can also assimilate DMSP from the surrounding environment [31,32,33], and that this phytoplanktonic uptake can match the magnitude of uptake by bacterioplankton [33,34]. A study using the temperate diatom *Thalassiosira weissflogii* recorded intracellular concentrations saturating at ~83 mM [32], underscoring the possibility that diatoms may form significant sinks and subsequent reservoirs for DMSP in the ocean. Both the presence of lyase activity in DMSP producers and DMSP uptake by non-producers suggests phytoplankton may play a larger role in marine sulfur ecology and DMS flux from the ocean than solely through the production of DMSP.

In this study, we aimed to better understand the phytoplankton sources and sinks of DMSP in Australian temperate waters. Through a whole phytoplankton community DMSP enrichment incubation, we sought to quantify the uptake of DMSP by the entire microbial community, providing an estimation of the demand for sulfur in these waters. Next, to differentiate the responses between microbial size classes and gain detailed insight into who the key sinks and sources might be, we fractionated the community into three size classes and enriched each fraction with DMSP, with the aim of uncovering the dominant sinks for dissolved DMSP in the waters off Australia’s eastern coastline.

## 2. Materials and Methods

### 2.1. Water Collection and Pre-Treatment

**Figure 1:**
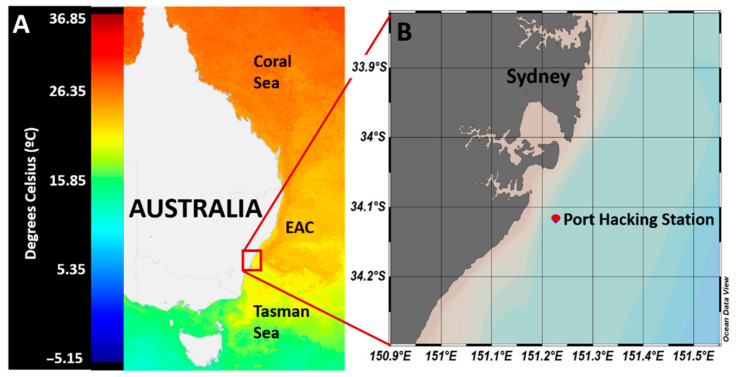
**Location of the sampling site.** (**A**) Satellite image of the sea surface temperature of the east coast of Australia on 14 April 2019 showing the location of Sydney basin and the position of the EAC relative to the Tasman and Coral Seas (modified from IMOS [38]). (**B**) Magnified inset image showing the location of Port Hacking station. Generated with Ocean Data View software [39].

Incubation experiments were conducted using surface (5 m) seawater samples collected from the Port Hacking (PH) oceanographic time-series station (−34.1192° S, 151.2267° E), located 30 km south of Sydney, Australia, and 8 km offshore (Figure 1). These waters are characterized by the complex interplay of oceanographic features; the East Australian Current (EAC), which originates in the Coral Sea, brings warm, oligotrophic waters south towards the Tasman Sea [35] before separating from the coast at 31–32° S [36], where the bulk of the water moves east towards New Zealand while the remainder continues south, breaking up into warm and cold core eddies (Figure 1A). Being at the tail end of the EAC means that the waters off the coast of the Sydney region are highly dynamic in nature with a mix of EAC and Tasman Sea influences [37]. Using these waters, two separate incubation experiments were conducted. The first of these involved a whole water incubation to investigate the uptake of DMSP by the natural marine microbial community while in the second, an incubation of the fractioned community was performed to quantify DMSP uptake in different phytoplankton size groups. Samples were collected in Niskin bottles using a CTD rosette on the 8 April 2019, and transported back to the laboratory in dark carboys for immediate processing, which occurred 4–6 h later. Seawater was first filtered through a 210-µm mesh to remove large grazers, before being sub-sampled into bottles for whole water incubations or size fractionated via serial filtration for the fractionated incubation experiments.

### 2.2. Incubation Set-Up for Whole Water and Size Fractionated Experiments

To study the uptake of DMSP by the natural microbial community, 10 L of seawater was transferred to polycarbonate bottles (4× controls, 4× + DMSP (20 nM final concentration)) with no headspace. An additional 2 bottles were amended with glutaraldehyde (1% final concentration) and DMSP (20 nM final concentration) to control for any passive uptake. Bottles were then closed with screw caps, shaken gently, and incubated for 17 h at 22.2 °C. Light was supplied by fluorescent bulbs at an irradiance of 100 µmol photons m^−2^ s^−1^ on a 12:12 light: dark cycle. Each bottle was subsampled in triplicate for total, dissolved, and particulate DMSP at three time points (T0 h, T5 h, and T17 h). Sub-samples were also taken for cell counts by flow cytometry and DMSP lyase activity (DLA) at the outset of the experiment (T0 h). For DLA determination, 600 mL was filtered first through a GF/C filter (nominal pore size 1.2 μm) for determination of phytoplanktonic-dominated DMSP lyase activity (DLAp), and then 300 mL of the phytoplankton-free filtrate was filtered through a 0.22-μm polycarbonate filter for the predominantly bacterial DLA component (DLAb). Both filters were placed into cryotubes, flash frozen in liquid nitrogen, and stored in −80 °C until analysis.

To study the uptake of DMSP by the different size classes of the microbial community, 20 L of seawater was size fractionated via gentle serial filtration using 8-, 3-, and 0.22-μm polycarbonate filters. During filtration, cells were gently re-suspended above the filter using a pipette and then washed with F/20 media for complete resuspension of the particulate matter before transferring into 400 mL of F/20 media. Media was added to ensure no nutrient limitation during overnight incubation (22.2 °C, 70 r.p.m, ~55 µmol photons m^−2^ s^−1^, 12:12 h light: dark cycle). The next day, the size fractioned community was split into control and + DMSP (100 nM final concentration) treatments (*n* = 4). Subsampling for total (whole water), dissolved (gravity filtered through GF/F filter), and particulate DMSP (cells obtained on filter) was carried out at three time points (T0, T3, and T6 h). At the final time point (T6 h), subsamples for chlorophyll *a* were also taken.

### 2.3. Quantification and Identification of Microbial Community

Populations of *Prochlorococcus*, *Synechococcus*, and picoeukaryotes were discriminated using side scatter (SSC) and red and orange fluorescence [40] and quantified using a Beckman Coulter Inc flow cytometer (Brea, CA, USA). Samples for bacterial analysis were stained with SYBR Green I nucleic acid stain (1:10,000 final dilution; Invitrogen) and populations were discriminated according to green fluorescence and side scatter properties [40,41,42]. Data were analyzed using CytExpert software v2.5 (Beckman Coulter Inc., Brea, CA, USA). Phytoplankton community composition, cell size, biovolume, and macronutrient concentrations were obtained from the Integrated Marine Observing System (IMOS) curated Australian Ocean Data Network (AODN) portal (https://portal.aodn.org.au/ accessed on 4 April 2021).

### 2.4. Sulfur Chemistry

Total DMSP (DMSPt), dissolved DMSP (DMSPd), and particulate DMSP (DMSPp) were quantified as total DMS after conversion with 100 mg of NaOH and measured using a gas chromatograph (GC-2010 Plus, Shimadzu, Kyoto, Japan) coupled with a flame photometric detector (FPD). Samples were purged with He (60 mL/min for 4 min) while cryo-trapped in liquid N_2_ and subsequently eluted onto a capillary column (DB-1, Agilent; injector: 120 °C, column: 110 °C, FPD: 130 °C, column flow: 2.1 mL min^−1^). Samples with high concentrations of DMSP (>500 pmol) were analyzed via direct injection of 500 µL of headspace (column flow: 3.66 mL min^−1^, FPD: 160 °C). Then, DMS was detected by the sulfur-specific detector in the GC, which was set up in the log mode (Log response vs. Log concentration) to obtain a linear response, as the emission intensity of sulfur is proportional to the square of the concentration. The DMS peak area was integrated against a calibration curve (R^2^ > 0.99) made of fresh DMS standards prepared from DMSP (PESTANAL, Sigma-Aldrich, Castle Hill, NSW, Australia) that were lysed to DMS with NaOH [43] and injected into the GC with the same injection mode as the samples. All DMSP data were normalized to Chl *a*.

DMSP lyase activity (DLA) was determined according to the methods of Harada et al. [44]. Briefly, each filter was defrosted and placed in a 14-mL glass vial with 1 mL of Tris buffer (pH = 8) and capped with a rubber stopper, vortexed for 10 s, and incubated for 20 min at room temperature prior to the addition of DMSP in close to substrate-saturated amounts (5 mM final concentration) and crimped immediately. The vial was then vortexed for 10 s and 5 sequential injections of headspace (500 µL for phytoplankton and 100 µL for bacteria) were loaded into the GC-FPD by direct injection throughout 30 min with approximately one injection every 5 min and the exact time of injections recorded. A blank containing a clean filter in 1 mL of Tris buffer (pH = 8) was run following the same procedure as for the samples to account for any background DMS production. The rate of DMSP lysed to DMS was then calculated by the slope of the linear increase of DMS concentration over the ~30 min of the analysis [45].

### 2.5. Data Analysis

Differences in the DMSPt, DMSPd, and DMSPp concentrations over time and between treatments were analyzed using a resemblance matrix based on the Euclidean distance and two-factor Permutational Multivariate Analysis of Variance (PERMANOVA) in a nested design, with pair-wise comparisons at each time points. These analyses were performed using PRIMER v6 (Primer-E Ltd., Plymouth, UK) statistical package [46] with PERMANOVA + module [47]. To test for differences between treatments for DLA and DMSPp:Chl a, data were checked for homogeneity of variance by a Levene’s test and analyzed using one-way Analysis of Variance (ANOVA) using the statistical package SPSS v.24 (IBM Statistics, Sydney, Australia). Pearson correlations between DMSP concentrations were performed using R [48].

## 3. Results

### 3.1. Characteristics of Initial Water Masses

At the time of this experiment, Port Hacking seawater was characterized by low nutrient concentrations, with silicate concentrations of 0.5 µM, phosphate and ammonium concentrations of 0.09 µM, and no detectable level of nitrate (Table 1). Dimethylsulfide (DMS), DMSPt, and DMSPd concentrations in the initial water sample were 1.51 ± 0.06, 16.4 ± 1.14, and 1.76 ± 0.65 nM, respectively (Table 1). DMSP lyse activity was 2281 ± 205 nM h^−1^ for the large fraction (phytoplankton dominant) and 3347 ± 168 nM h^−1^ for the smaller fraction (bacterial).

Reflective of nutrient-poor waters, flow cytometric counts of the microbial community revealed relatively low abundances of *Synechococcus* (1.35 ± 0.01 × 10^5^ cells mL^−1^), *Prochlorococcus* (4.89 ± 0.58 × 10^4^ cells mL^−1^), heterotrophic bacteria (1.37 ± 0.34 × 10^5^ cells mL^−1^), and picoeukaryotes (2.01 ± 0.40 × 10^4^ cells mL^−1^) (Table 2). For the phytoplankton community, 16 taxa were identified across several phytoplankton classes, including: four centric diatoms, one pennate diatom, one cyanobacteria, four dinoflagellates, a chlorophyte, a prymnesiophyte, and a silicoflagellate. Several smaller unidentified dinoflagellates, flagellates, and cryptophytes were also abundant (Table 3).

### 3.2. Whole Community Incubation Experiment

**Figure 2:**
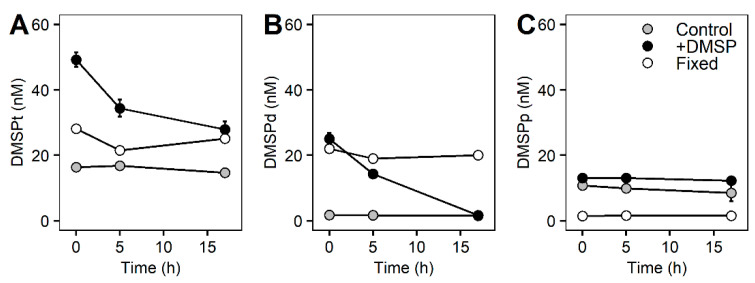
**DMSP concentrations of the whole marine microbial community over 17 h.** Concentrations of DMSPt (**A**), DMSPd (**B**), and DMSPp (**C**) over 17 h for control samples (light grey circles), DMSP-enriched samples (black circles), and fixed samples (white circles).

**Figure 3:**
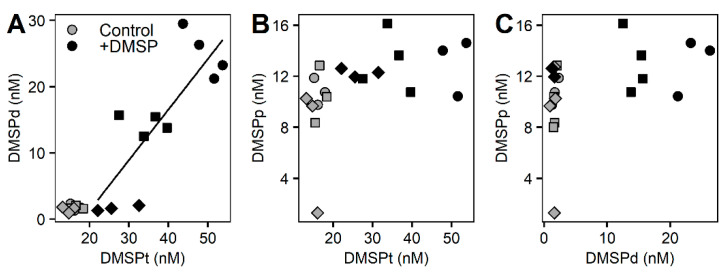
**Correlations between the different DMSP concentrations.** Control samples (light grey) and enriched DMSP samples (black) for time 0 h (circles), 3 h (squares), and 6 h (diamonds) are represented for DMSPd vs. DMSPt (**A**), DMSPp vs. DMSPt (**B**), and DMSPp vs. DMSPd (**C**). Significant correlation: (**A**) r^2^ = 0.668, *p* = 0.002.

Concentrations of DMSP for the whole community incubation experiment responded differently between control and +DMSP samples (Figure 2). The control samples had constant values for all sulfur compounds over the course of the experiment with concentrations of ~15 nM of DMSP total (Figure 2A), out of which ~2 nM occurred within the dissolved fraction and ~10 nM was in the particulate fraction (Figure 2B,C). Samples enriched with DMSP showed a significant decrease in DMSPt over time from 49.2 to 27.9 nM (PERMANOVA, Pseudo-F = 10.476, PMC = 0.001), representing a loss of ~43% over 17 h (Figure 2A). There was also a significant decline in DMSPd from 25.06 to 1.65 nM (PERMANOVA, Pseudo-F = 34.884, PMC = 0.001), equating to an ~93% loss, which over 17 h is a rate of disappearance of 33 nM d^−1^ (Table 4). Interestingly, DMSPp remained constant at ~12 nM (Figure 2C). As expected, samples fixed with glutaraldehyde showed constant DMSP concentrations over the course of the experiment (Figure 2). We found a significant positive correlation (Pearson correlation, r^2^ = 0.668, *p* = 0.002) between total and dissolved DMSP for the +DMSP treatment only, showing a clear loss in both fractions over time, revealing that the loss of DMSPt is largely due to a decrease in the dissolved fraction (Figure 3A). No relationships were detected between DMSPp and the other two sulfur fractions (Figure 3B,C).

### 3.3. Fractioned Community Incubation Experiment

**Figure 4:**
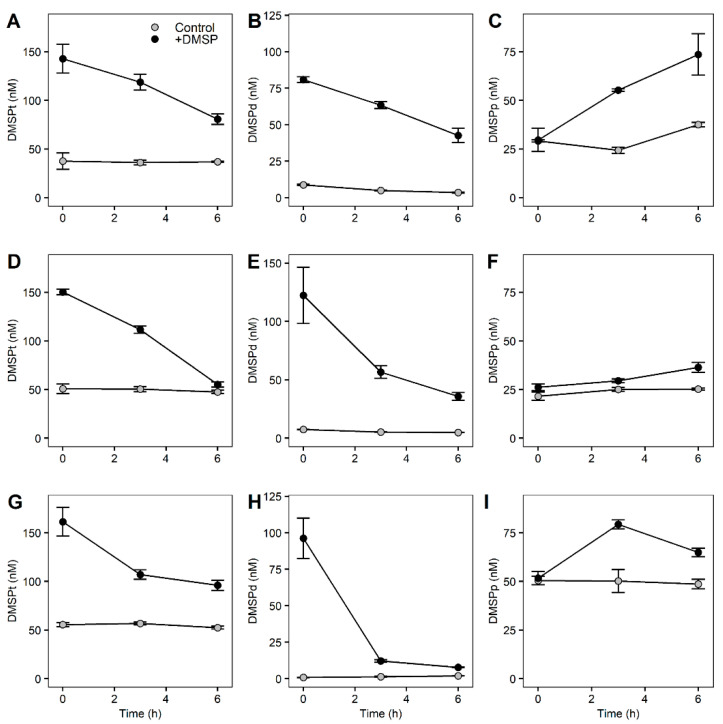
**DMSP concentrations of fractionated marine microbial community over 6 h.** Top: >8 µm fractioned samples (DMSPt (**A**), DMSPd (**B**), DMSPp (**C**)), middle: 3–8 µm fractioned samples (DMSPt (**D**), DMSPd (**E**), DMSPp (**F**)), bottom: 0.22–3 µm fractioned samples (DMSPt (**G**), DMSPd (**H**), DMSPp (**I**)) for control samples (light grey) and +DMSP samples (dark grey).

Concentrations of DMSP for the fractionated community showed similar responses across the different fractions with decreasing concentrations of DMSPt and DMSPd and increasing concentrations of DMSPp for the +DMSP samples (Figure 4). In the controls of the largest fraction (>8 µm), DMSPt remained stable at around 38 nM while DMSPd decreased significantly from 8.77 to 3.44 nM (paired *t*-test, t06 = 5.7967, *p* = 0.003), and DMSPp increased (paired *t*-test, t06 = 6.3069, *p* = 0.002) from 29.14 to 37.51 nM (Figure 4A–C). In the +DMSP samples, DMSPt decreased from 142.84 to 89.21 nM (paired *t*-test, t06 = 3.1152, *p* = 0.019), as did DMSPd from 80.87 to 42.62 nM (paired *t*-test, t06 = 5.5153, *p* = 0.003) while there was an increase in DMSPp from 29.59 to 73.58 nM (paired *t*-test, t06 = 3.4614, *p* = 0.016) (Figure 4A–C).

**Table 4:** Relative loss/gain and rate of DMSP for each size fraction and treatment for DMSPt, DMSPd and DMSPp. This is a numerical summary of data presented in Figure 4. Relative loss/gain was calculated as % of DMSP lost/gained relative to its initial concentration (i.e., Δ Concentration/initial concentration × 100). Loss/gain rates were determined as the change in concentration over time and obtained from the slope of the linear regression. Samples with no significant (*p* > 0.05) DMSP loss or gain between initial and final time have been omitted and are indicated with a dash. Samples that lose DMSP over time are indicated with negative values and samples that gain DMSP over time are indicated with positive values. Data show mean +/− SD and *n* = 3 *.

		*DMSP Total*		*DMSP Dissolved*		*DMSP Particulate*	
Fraction (µm)	Treatment	Rel. Loss/Gain (%)	Loss/Gain Rate (nM h^−1^)	Rel. Loss/Gain (%)	Loss/Gain Rate (nM h^−1^)	Rel. Loss/Gain (%)	Loss/Gain Rate (nM h^−1^)
>8	Control	-	-	−62. 9 ± 6.8	−0.9 ± 0.02	+31.1 ± 7.4	+1.5 ± 7.3
>8	+DMSP	−41.8 ±16.2	−10.4 ± 5.4	−46.9 ± 12.6	−6.4 ± 1.9	+143 ± 166	+0.3 ± 4.6
>3	Control	-	-	−40.2 ± 3.8	−0.4 ± 0.07	-	-
>3	+DMSP	−63.3 ± 2.3	−15.8 ± 0.3	−46.9 ±12.6	−6.4 ± 1.9	+41.5 ± 30.3 *	+1.6 ± 1.5
>0.22	Control	-	-	-	-	-	-
>0.22	+DMSP	−39.1 ± 12.9	−10.9 ± 5.4	−91.6 ± 1.9	−14.7 ± 4.0	+27.3 ± 22.8	+2.2 ± 1.6

In the control samples of the medium fraction (>3 µm), there was no change in DMSPt over time while DMSPd decreased (paired *t*-test, t06 = 11.062, *p* = 0.001) from 7.38 to 4.84 nM (Figure 4D,E). There was no change in DMSPp over the 7 h (Figure 4F). Similar to the largest fraction, the +DMSP samples showed a decline in DMSPt from 150.24 to 55.20 nM (paired *t*-test, t06 = 3.2491, *p* = 0.02) and DMSPd from 122.39 to 35.80 nM (paired *t*-test, t06 = 5.5153, *p* = 0.009), and an increase (paired *t*-test, t06 = 5.1533, *p* = 0.01) in DMSPp from 26.17 to 36.35 nM (Figure 4D–F).

In the smallest fraction (>0.22 µm), there were no significant changes in any DMSP concentrations over time for controls (Figure 4G–I). However, in the DMSP-enriched samples, there was a significant decline in DMSPt from 161.27 to 95.99 nM (paired *t*-test, t06 = 4.8277, *p* = 0.01) and DMSPd from 96.23 to 7.72 nM (paired *t*-test, t06 = 14.659, *p* = 0.001) while DMSPp increased significantly (paired *t*-test, t06 = 4.3163, *p* = 0.007) from 50.19 to 66.43 nM (Figure 4G–I).

We observed significant losses in DMSPt in all fractions of the community when enriched with DMSP (Figure 4; Table 4), indicating that catabolism of DMSP to DMS or MeSH occurred in all size fractions when DMSP was available in high concentrations. Loss rates varied within fractions, whereby the >3 µm fraction had the highest DMSPt loss rate of 15.840 nM h^−1^ compared to 10.377 and 10.880 nM h^−1^ for the >8 and >0.22 µm fraction, respectively (Table 4). All fractions showed a loss of DMSPd at a similar rate to DMSPt, suggesting that most of the loss measured from the total pool was from the dissolved fraction. The accumulation of DMSPp, which was significant in all three fractions, occurred at a slower rate (0.355–2.201 nM h^−1^) than the other compounds (Table 4). However, the rate of DMSPp gain shown in Table 4 does not consider the amount of DMSP taken up and immediately catabolized by the cell. Therefore, rather than indicating the rate of DMSP taken up by the cell, it indicates the rate in which DMSP accumulates into the cells and as DMSPp was increasing over time, it suggests that the uptake of DMSP was faster than its degradation.

**Figure 5:**
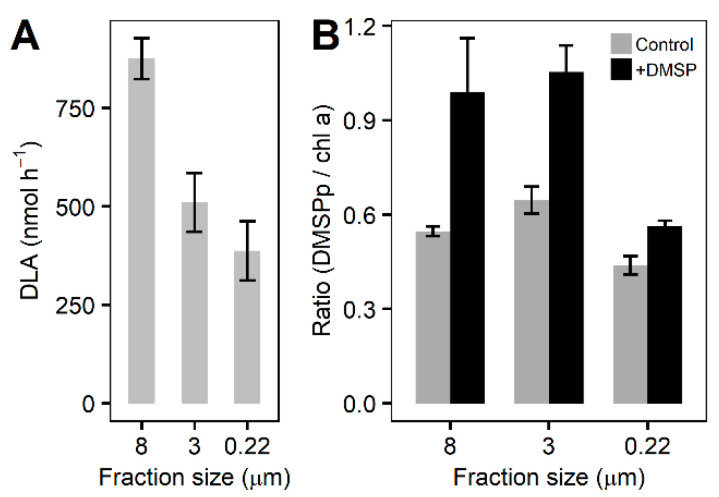
**DLA and DMSPp at the final time point.** (**A**) DLA for each fraction of the community at the final time point. (**B**) DMSPp concentrations normalized to chlorophyll *a* for control samples (light grey bars) and +DMSP samples (dark grey bars) for each fraction of the community at the final time. Data represent the mean ± standard deviation (*n* = 4). ANOVA, 8 µm: F = 14.748, *p* = 0.012; 3 µm: F = 14.777, *p* = 0.012; 0.22 µm: F = 12.818, *p* = 0.023.

The rates of DLA were highest (875 ± 104 nmol h^−1^) in the largest size fraction (ANOVA, F = 10.313, *p* = 0.008), followed by the >3 µm (510 ± 149 nmol h^−1^) and smallest (>0.2 µm; 387 ± 150 nmol h^−1^) fractions (Figure 5A). Chlorophyll *a* normalized DMSPp was significantly higher in the +DMSP samples after 6 h (ANOVA, 8 µm: F = 14.748, *p* = 0.012; 3 µm: F = 14.777, *p* = 0.012; 0.22 µm: F = 12.818, *p* = 0.023) compared with the controls for each fraction (Figure 5B). The percentage of DMSP taken up by each fraction with respect to the control showed the two larger fractions (>8 and 3–8 µm) to almost double in DMSPp concentration, representing an 81% and 63% increase, respectively (Figure 5B). For the >0.22 µm fraction, DMSPp increased by approximately 30%.

**Figure 6:**
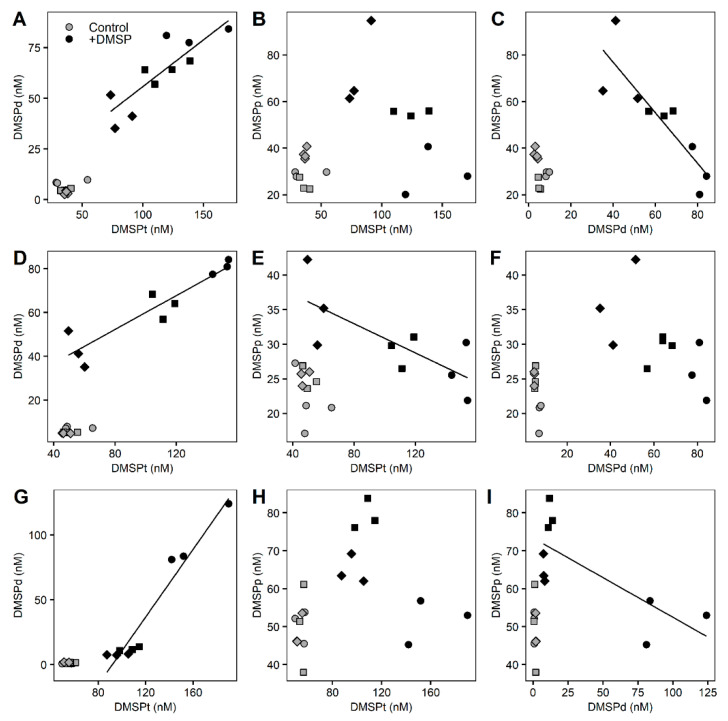
**Correlations between the different DMSP concentrations for the fractionated marine microbial community**. Control samples (light grey) and enriched DMSP samples (black) for time 0 h (circles), 3 h (squares), and 6 h (diamonds) are represented. Top row: >8 µm fraction (**A**–**C**), middle row: 3–8 µm fraction (**D**–**F**), bottom row: 0.22–3 µm fraction (**G**–**I**). Significant correlations: (**A**) r^2^ = 0.722, *p* = 0.0019; (**C**) r^2^ = 0.746, *p* = 0.0027; (**D**) r^2^ = 0.861, *p* = 0.0003; (**E**) r^2^ = 0.565, *p* = 0.0195; (**G**) r^2^= 0.942, *p* = 0.01; (**I**) r^2^ = 0.551, *p* = 0.02.

Significant correlations occurred between DMSP compounds in the +DMSP samples across all size fractions (Figure 6). In the >8 µm fraction, DMSPd concentrations showed a positive correlation with DMSPt (Pearson correlation, r^2^ = 0.722, *p* = 0.0019) and a negative correlation (Pearson correlation, r^2^ = 0.746, *p* = 0.0027) with DMSPp while no relationship was detected between DMSPp and DMSPt (Figure 6A–C). For the >3 µm fraction, DMSPt was correlated positively with DMSPd (Pearson correlation, r^2^ = 0.861, *p* = 0.0003) and negatively with DMSPp (Pearson correlation, r^2^ = 0.565, *p* = 0.0195) while there was no relationship between DMSPp and DMSPd (Figure 6D–F). As with the largest fraction, within the smallest (>0.22 µm) fraction, there was a positive correlation between DMSPt and DMSPd (Pearson correlation, r^2^= 0.942, *p* = 0.01) and a negative relationship between DMSPd and DMSPp (Pearson correlation, r^2^ = 0.551, *p* = 0.02), with no relationship detected between DMSPt and DMSPp (Figure 6G–I).

## 4. Discussion

Phytoplankton dynamics are closely linked to climate, and changes in ocean conditions derived from climate change are substantially altering phytoplankton biogeography, abundance, and phenology, favoring species best adapted to changing conditions [49]. Over the last few decades, the EAC has strengthened, intruding further southward, resulting in warmer temperatures, higher salinity, and lower silicate and dissolved oxygen concentrations becoming more typical at PH [50]. Changes in the phytoplankton composition have also been observed, with an emerging dominance of tropical phytoplankton species such as *Trichodesmium erythraeum* and *Bacteriastrum* spp. frequently replacing many temperate taxa [51]. In this study, we observed the influence of the East Australian Current (EAC) on the waters sampled, including a low nutrient signal—with values within ranges of previous EAC studies [52,53]—and a high abundance of *Trichodesmium* sp. An ecologically important diazotrophic cyanobacterium, *Trichodesmium* spp. dominate nitrogen-poor waters [54]. It is difficult to say if the presence of *Trichodesmium* sp. at PH is a consequence of climate-induced strengthening of the EAC because of the high variability in species abundance over seasonal, inter-annual, and inter-decadal time scales [55]. However, since it is suggested to be a DMSP producer [56,57] and has been one of the two dominant species at PH in the last two decades [51], it is likely that this taxon will play an increasingly important role in the microbial ecology and seasonal cycling of DMSP in the temperate waters of Port Hacking.

The total DMSP and DMS concentrations measured in this study closely match the annual averages of 25.7 ± 5.31 (DMSPt) and 2.7 ± 0.30 nM (DMS) for Port Hacking measured over a 2-year study and are consistent with, albeit slightly higher than, April DMS/P concentrations for Port Hacking [58]. The relatively high concentration of DMSP measured in the seawater during this study can be attributed to the generally high intracellular concentrations in the DMSP-producing species within the community, including dinoflagellates [59], *Phaeocystis* sp. [60], and *Trichodesmium* sp. [17,18], all of which were present in relatively high abundances. The low levels of DMS, however, suggest that despite the presence of *Phaeocystis*, which is known to cleave DMSP to DMS [25,30], the sulfur demand of the community was potentially greater than production [19], and that for DMS flux to increase in PH, there would need to be an injection of additional DMSPd into the water column. This was confirmed by our incubation experiments. The lack of change in the control incubations demonstrated stability in the production and consumption processes of DMSP by the PH microbial community. This stability could indicate that the sulfur demand of the community was not yet satisfied and that much of the DMSP was retained inside the cells to be used in one of its cellular roles [61]. Furthermore, it was clear that when enriched with DMSP, the microbial community took up most of the available DMSP rapidly, metabolizing the excess. Indeed, we observed a decline in both the total and dissolved DMSP over time while the particulate fraction remained constant, indicating that the additional DMSP available was taken up and rapidly transformed to DMS or MeSH and not retained within the cells [22]. Furthermore, the positive correlation between DMSPt and DMSPd for the +DMSP samples supports the idea that the excess DMSP was cleaved into DMS and lost to the atmosphere [22,62]. This response indicates that increases in the DMSPd pool in the ocean would likely result in an increased flux of DMS to the atmosphere.

The fractionation approach (experiment 2) allowed us to gain insight into how the DMSP sources and sinks were distributed within the microbial community of PH. As seen for the whole community, most of the DMSP in the control samples was retained within the particulate fraction, especially for the largest and smallest size classes. Similarly, concentrations of DMSPt remained constant over time in all fractions, indicating that the sulfur demand was close to satisfied and DMSP could be retained inside the phytoplankton cells to be used for cellular function [61]. Unlike the whole community incubation, all fractions when enriched with DMSP increased in DMSPp over time, indicating that the degradation of DMSP was slower than its uptake and allowed DMSP to accumulate inside the cells, a response also observed for natural marine microbial communities of the Great Barrier Reef [33] and in cultured *Thalassiosira weissflogii* [32,63]. The difference between the whole water and fractioned samples with respect to DMSPp may be attributed to the whole community incubations being conducted at lower cell densities with its microbial community intact, potentially consisting of many active DMSP degraders, compared with the higher microalgal cell densities used in the fractionated experiments contributing to a more pronounced change, given that the majority of the microbial community would have been filtered out, at least in the two largest size fractions. These data indicate that DMSP was being taking up by non-producers, helping to meet sulfur demands, and that once sulfur demands were met, the excess DMSP was being catabolized to produce DMS or removed from the system via photo-oxidation.

The cell size-dependent accumulation of DMSP observed in this study may be attributable to the greater volume of larger cells, such as in centric diatoms, which have vacuoles that may act as storage sites for osmolytes and other compounds [64]. However, independent of the uptake capacity, this study has provided evidence that phototrophs, present in the largest size fractions, form major DMSP sinks in the temperate waters of eastern Australian shelf waters, and support the hypothesis that autotrophic phytoplankton take up significant amounts of DMSP [32,33,34]. It is important to note that the term sink here refers to the active uptake of DMSP by a photoautotroph, with no indication or assumption about its retention time or fate. Previous work has shown that accumulation of DMSP by non-producers is likely to be transient, with accumulation and re-release or metabolization occurring within 24 h [33]. The duration of both experiments in this study was less than 24 h and in both instances no data on re-release or retention time were obtained. Given that non-DMSP-producing phytoplankton take up DMSP, it is plausible that they may also have the capacity to metabolize it. However, limited research has been carried out on DMSP degradation by phytoplankton and to date, there is no evidence for any demethylation pathway in phytoplankton, leaving the door open for further research.

Of the major phytoplankton taxa that made up the community, the high producers of DMSP (HiDP) were the haptophyte *Phaeocystis* sp. and dinoflagellates. *Trichodesmium*, while a DMSP producer, is reported to have low intracellular DMSP concentrations with values of 0.05 mM [18], but given its abundance at the time of sampling, its contribution to the DMSP pool was possibly significant. Dinoflagellates have reported intracellular DMSPp concentrations ranging from 45.5–124.6 mM [59] and given that they too were present, albeit in lower abundances for the larger cells (3.797% cells L^−1^), they were likely major contributors to the particulate DMSP concentrations. The most prolific DMSP producer (concentrations between 121 and 358 mM) present in samples was non-colonial *Phaeocystis* sp. [59], but given their small size and moderate relative abundance, they may not have had as much of an influence on the sulfur production at PH at this time. *Phaeocystis* spp. is the only taxa within our study that is known to cleave DMSP to DMS, with previous measurements indicating that species within this genus can produce up to 3.05 nmol DMS min^−1^ [30]. Interestingly, however, our size-sorted results suggested that all fractions cleaved DMSP to DMS, although at different rates, which points to two likelihoods: (1) that there were DMSP-cleaving bacteria present in each fraction, whereby bacterial abundance may have increased during incubation and may have differed between treatments, and/or (2) that some taxa possess the yet unknown capability to lyse DMSP into DMS.

Of the proposed non/low-DMSP-producing taxa, the diatoms likely formed the major DMSP sink for PH waters at the time of sampling, together with the picoeukaryotes that were present in high abundance. Further research is needed, however, to establish whether these non- or low-DMSP-producing phototrophs metabolize the DMSP, and whether it is used by the cells to adapt to environmental change. Importantly, this study adds to a growing body of evidence showing that the phototrophic microbial community contains novel players in coastal sulfur cycling, whose roles have been previously overlooked.

## 5. Conclusions

Dimethylsulfoniopropionate (DMSP) is a key chemical compound that underpins many ecological interactions and metabolic processes within marine microbial communities. The uptake and subsequent degradation of DMSP has been well studied in bacteria, but only a few experiments have studied its uptake and utilization in phytoplankton. This study evaluated the uptake of DMSP by phytoplankton and bacteria in natural seawaters of a temperate location in east Australia. We found that when the whole community was enriched with DMSP, concentrations of DMSPd and DMSPt declined over time, indicating that DMSP was being taken up and utilized by the microbial community. However, studying the microbial community as a whole revealed only a small part of the sulfur dynamics. Incubations of different size classes of the community showed an increase in DMSPp with enrichment, indicating that phototrophic microbes from all size fractions took up and accumulated available DMSPd. The results from this study support previous work showing that both bacteria and phytoplankton take up dissolved DMSP from the environment and provide evidence that many large non-DMSP-producing species act as important DMSP reservoirs in temperate waters

## Figures and Tables

**Table 1:** Environmental parameters of initial seawater. Salinity, chlorophyll a, silicate, nitrate, phosphate, and ammonium concentrations at the initial sampling time point obtained by IMOS [38]. Dimethylsulfide (DMS), total DMSP (DMSPt), DMSPd, DLAb, and DLAp concentrations of the initial seawater.

Parameter	
Salinity (psu)	35.4
Chlorophyll *a* (µg L^−1^)	0.306
Silicate (µM)	0.5
Nitrate (µM)	nd
Phosphate (µM)	0.09
Ammonium (µM)	0.09
DMS (nM)	1.51 ± 0.06
DMSPtotal (nM)	16.4 ± 1.41
DMSPdissolved (nM)	1.76 ± 0.65
DLAphyto (nM h^−1^)	2281 ± 205
DLAbacto (nM h^−1^)	3347 ± 168

**Table 2:** Flow cytometric counts of the initial microbial community.

Microbial Composition	
*Synechococcus* (cells × 10^5^ mL^−1^)	1.35 ± 0.01
*Prochlorococcus* (cells × 10^4^ mL^−1^)	4.89 ± 0.58
Heterotrophic Bacteria (cells × 10^5^ mL^−1^)	1.37 ± 0.34
Picoeukaryotes (cells × 10^4^ mL^−1^)	2.01 ± 0.40

**Table 3:** Composition of the phytoplankton community, their cell biovolume, fraction, and DMSP production status. Phytoplankton were quantified and identified by IMOS [38]. The smallest fraction (bottom section of table), consisting of mostly flagellates and cryptophytes, were quantified in the picoeukaryote fraction of flow cytometry data. Hi and Lo DMSP-producing classification is taken from McParland et al. [17]. In cases where the species does not match, classification has been matched to genus. nd = no data.

Group	Species	Cells L^−1^	Biovolume (µm^3^ L^−1^)	Size Fraction	DMSP Producer (Hi/Lo)
Centric diatoms	*Skeletonema* spp.	164	67,453	3–8, >8 µm	Lo
	*Climacodium* spp.	235	79,516,888	>8 µm	nd
	*Dactyliosolen* spp.	211	10,370,566	>8 µm	nd
	*Chaetoceros* spp.	47	17,699	3–8, >8 µm	Lo
Pennate diatoms	*Cylindrotheca closterium*	915	183,099	>8 µm	Lo
Cyanobacteria	*Trichodesmium* spp.	3286	1,881,635	>8 µm	Lo
Dinoflagellates	*Gyrodinium* spp. (20–40 µm)	23	184,366	>8 µm	Lo
	*Tripos* ^ *candelabrum*	23	1,260,876	>8 µm	Hi
	*Tripos* ^ *macroceros*	23	1,622,417	>8 µm	Hi
	*Protoperidinium* spp.	23	1,812,388	>8 µm	Hi
Prymnesiophyte	*Phaeocystis* sp.	854	96,541	3–8 µm	Hi
Chlorophyte	Prasinophyte	4268	1,117,367	3–8 µm	Lo *
Silicoflagellate	*Dyctyocha fibula*	23	96,024	>8 µm	nd
Other	Unid. Dinoflagellate (<10 µm)	7682	2,316,973	3–8, >8 µm	Hi *
	Flagellates <10 µm	168,161	11,006,069	3–8, >8 µm	nd
	Cryptophytes <10 µm	18,779	14,749,249	>8 µm	nd

* for unidentified groups, the DMSP production level is assumed based on the phytoplankton grouping. ^ Listed as *Ceratium* in McParland et al. [16].

## Data Availability

The data sets generated during and/or analysed during the current study are available from the corresponding author on reasonable request. PH environmental and taxonomic data are available from the IMOS Australian Ocean Data Network (AODN) portal (https://portal.aodn.org.au/ accessed on 4 April 2021).
